# Vitamin D and Kawasaki disease

**DOI:** 10.3389/fphar.2026.1851161

**Published:** 2026-06-02

**Authors:** Dan-Dan Yang, Yuan-Yuan Zeng

**Affiliations:** Department of Pharmacy, Children’s Hospital of Nanjing Medical University, Nanjing, China

**Keywords:** coronary artery lesionsdisease, immunoglobulin resistance, inflammation, kawasaki disease, vitamin D

## Abstract

**Background:**

Vitamin D deficiency has been widely reported worldwide. Vitamin D is essential for mineral homeostasis and skeletal health, and is also associated with numerous extra-skeletal diseases, including cancer, autoimmune diseases, psychosis, allergies and cardiovascular disease. Recent evidence suggests that vitamin D deficiency might be a risk factor for immunoglobulin resistance in Kawasaki disease (KD), and that levels of 25-hydroxyvitamin D_3_ (25(OH)D_3_) could predict the risk of coronary artery disease in KD patients. These findings have sparked growing interest in exploring the mechanistic and clinical links between vitamin D and KD pathogenesis. However, the relationship between vitamin D levels and KD remains incompletely understood, warranting further comprehensive investigation.

**Mechanisms and evidence reviewed:**

Previous studies found that proinflammatory cytokines, such as tumor necrosis factor (TNF)-α and interleukin (IL)-6, are elevated during the acute phase of KD and are associated with activation of vascular endothelial cells, which can lead to varying degrees of vascular wall inflammation. Vitamin D, a multifunctional prohormone with well-documented anti-inflammatory properties, may exert protective effects in KD by enhancing endothelial function, reducing coronary artery damage risk, and inhibiting platelet activity to alleviate fibrinolysis, thrombosis, and inflammation.

**Conclusions:**

This study updates the current understanding of the association between serum vitamin D levels and KD, explores potential regulatory mechanisms underlying this relationship, and identifies critical knowledge gaps to guide future cohort studies and clinical trials in KD research. Early clinical data indicate that adjunctive vitamin D may modulate inflammatory responses in acute KD, but whether supplementation reduces intravenous immunoglobulin (IVIG) resistance or prevents coronary artery lesions remains unproven and requires prospective investigation.

## 1 Introduction

First described in Japan 59 years ago, Kawasaki disease (KD) is now a global multisystem disorder (Kawasaki, 1967). It is an acute self-limited vasculitis of unknown etiology that can cause coronary artery lesions (CALs), including dilatation, aneurysms, and stenosis in children, and it is the leading cause of acquired heart disease among children in developed countries (Jone et al., 2024). The annual incidence of KD among children aged 0–4 years per 100,000 population varies considerably worldwide: 319.6 in Japan (2015–2016), 170.9–194.9 in Korea (2014), 71.9–110.0 in China, 49.4 in Hawaii, and 18.1–21.3 in the USA (Fukazawa et al., 2020). Incidence was highest in Asian children, especially those of Japanese descent. Previous studies showed that inflammatory responses during the acute phase of KD can lead to varying degrees of vascular wall inflammation (Li et al., 2019; Yamaji et al., 2019; Ferdosian et al., 2021). Vitamin D is a multifunctional prohormone proposed to exert widespread effects in humans (Ellison and Moran, 2021). The active form, 1,25-dihydroxyvitamin D_3_ (1,25-(OH)_2_D_3_), signals via the vitamin D receptor (VDR), which is expressed in nearly every tissue, including the heart and blood vessels. Recent evidence indicates that vitamin D deficiency might be a risk factor for intravenous immunoglobulin (IVIG) resistance in KD (Visuddho et al., 2024). IVIG resistance in KD patients require additional treatment (Wang et al., 2024), and represents a higher risk of developing CALs (Zheng et al., 2021). Some studies suggested that adjunctive 1,25-(OH)_2_D_3_ can modulate the inflammatory response in KD vasculitis (Zhang et al., 2024), and that 25-hydroxyvitamin D3 (25-(OH)D_3_) levels may predict CALs risk in mouse model of KD (Chen et al., 2025). However, the potential mechanisms underlying this association remain unexplored. In this study, we update the current evidence on the association between serum vitamin D levels and KD, and explore the potential regulatory mechanisms by which vitamin D modulates KD-related inflammation and vascular injury. Our focus includes key pathways (nuclear factor kappa-B (NF-κB), toll-like receptor 4 (TLR4), P53/extracellular signal-regulated kinase (ERK)) and cellular processes (endothelial progenitor cells (EPCs) and platelet biology). We also identify critical knowledge gaps to inform future cohort studies and clinical trials in KD research.

## 2 Information sources and search strategy

PubMed, Embase, the Cochrane Library, Web of Science, China National Knowledge Infrastructure (CNKI), and Wanfang database were systematically searched from their establishment date to 31 December 2025 to explore the relationship between vitamin D status and KD. A combination of controlled vocabulary (such as MeSH terms in PubMed and Emtree terms in Embase) and free-text keywords was employed, with search terms centered on three core concepts: “vitamin D”, “Kawasaki Disease”, and related outcomes (such as “inflammation”, “immune response”, and “cytokines”). Studies were included if they met the following criteria: (1) Study design: Original research articles (including clinical trials, cohort studies, case-control studies, and experimental studies in animal models), systematic reviews, and meta-analyses focused on the relationship between vitamin D and KD; (2) Population: Pediatric patients diagnosed with KD based on standard clinical criteria, or animal models established to mimic KD pathophysiology; (3) Exposure/intervention: Studies investigating vitamin D status (e.g., serum 25-hydroxyvitamin D levels), vitamin D supplementation, or the effects of vitamin D on KD-related outcomes; (4) Outcomes: Evaluation of clinical outcomes (e.g., coronary artery lesion development), inflammatory markers (e.g., C-reactive protein, interleukin-6), immune cell function, or cardiovascular complications associated with KD; (5) Language: Articles published in English. Studies were excluded if they: (1) review articles, editorials, letters, or conference abstracts lacking original data or detailed analysis; (2) Included overlapping or duplicate data; (3) Failed to report outcomes related to vitamin D’s effects on KD or its complications. This review is a narrative synthesis of the literature. A systematic search strategy was employed only for the clinically relevant sections. Formal quality assessment and quantitative synthesis (i.e., meta-analysis) were not performed for all cited studies.

The evidence cited in this review varies in strength. Higher-level clinical support comes from the Zhang et al. (2024) meta-analysis of 22 studies, the Zheng et al. (2021) meta-analysis on IVIG resistance and CALs, the Yang et al. (2020) nationwide pediatric cohort of over 460,000 children, the 2024 AHA Scientific Statement on KD, and the JCS/JSCS guideline; these sources form the basis for the review’s rationale, background, and principal conclusions. In contrast, results from single *in vitro* experiments, mouse models, and cross-disease extrapolations (for example, from congestive heart failure or non-KD vascular injury) are hypothesis-generating and warrant cautious interpretation. A detailed evidence summary with strength grading is provided in Supplementary Appendix S1 for readers’ reference.

## 3 Biological properties of vitamin D

Vitamin D is a group of fat-soluble compounds derived from cholesterol in animals (cholecalciferol, vitamin D_3_) and from ergosterol in plants and fungi (ergocalciferol, vitamin D_2_). Vitamin D_3_ is synthesized in the skin and is found in animal foods such as fish, whereas vitamin D_2_ occurs in fungi and plants. Research showed that vitamin D is a prohormone (Tuckey et al., 2019), that must be converted to its active form (Sirajudeen et al., 2019). Nearly all of vitamin D’s biological effects are believed to be mediated by its active metabolite (Janoušek et al., 2022). In hepatocytes, vitamin D_3_ or D_2_ is hydroxylated by microsomal hydroxylase to form 25(OH)D_3_, which has relatively low biological activity. Renal proximal tubular epithelial cells then convert 25(OH)D_3_ to calcitriol 1,25-(OH)_2_D_3_ via 1-α-hydroxylase (Lee et al., 2025). 25(OH)D_3_ serves as the primary circulating storage form of vitamin D, with a long half-life (∼3 weeks) and is widely recognized as the gold standard for assessing vitamin D nutritional status (Chinese Society of Osteoporosis and Bone Mineral Research, 2025), while 1,25(OH)_2_D_3_ represents the biologically active metabolite, tightly regulated by parathyroid hormone (PTH), fibroblast growth factor 23 (FGF23), and calcium levels, exerting its effects through binding to the VDR to modulate calcium homeostasis, immune function, and cellular differentiation (Adams and Hewison, 2010; Bikle, 2014). Almost every human tissue expresses VDR (Ricca et al., 2018). VDR belongs to the nuclear receptor family of ligand-activated transcription factors, and performs diverse biological functions (Khammissa et al., 2018). Activation of VDR by 1,25-(OH)_2_D_3_ modulates the expression of more than 1000 genes across multiple tissues (Zmijewski and Carlberg, 2020). Although Vitamin D is essential for mineral homeostasis and skeletal health, decades of research have linked vitamin D status to numerous extra-skeletal conditions, including cancer (Schömann-Finck and Reichrath, 2024), autoimmune diseases (Hahn et al., 2022), psychosis (Roy et al., 2021), allergies (Briceno Noriega and Savelkoul, 2021) and cardiovascular disease (Manson et al., 2019; Latic and Erben, 2020).

## 4 Vitamin D status among children

Vitamin D deficiency has been widely reported worldwide (Fiamenghi and Mello, 2021; Bajpai, 2022; Ganmaa et al., 2023). Serum 25(OH)D_3_ levels above 50 nmol/L are considered to be adequate (Bacchetta et al., 2022). However, a systematic literature review encompassing 195 studies from 44 countries and over 168,000 participants found that mean population-level 25(OH)D_3_ values varied considerably across the studies (range 4·9–136·2 nmol/L), with 37·3% of the studies reporting mean values below 50 nmol/L (Hilger et al., 2014). The review also found that mean 25(OH)D_3_ values in North America were significantly higher than those in Europe and the Middle East/Africa, while no significant difference was observed between North America and the Asia- Pacific region. No significant age-related difference was observed in global 25(OH)D_3_ concentrations among eligible study samples. However, in Asia, children and adolescents have significantly lower 25(OH)D_3_ levels than adults and the elderly. This difference appears to reflect particularly low 25(OH)D_3_ concentrations among Chinese children and adolescents, who also have low dietary calcium intake and limited sun exposure. A recent retrospective survey (Yang et al., 2020) of more than 460,537 children and adolescents from mainland China reported vitamin D deficiency (<30 nmol/L) and insufficiency (30–50 nmol/L) prevalences of 6.7% and 15.9%, respectively. The study also found that vitamin D status is worse among newborns and children presenting to the hospital in winter.

## 5 Mechanistic basis of the effect of vitamin D on Kawasaki disease

### 5.1 Anti-inflammatory effects of vitamin D on arterial endothelial cells

KD is an acute febrile vasculitis involving small- and medium-sized arteries, that can progress to coronary artery damage. Previous studies showed that several proinflammatory cytokines, including tumor necrosis factor (TNF)-α and interleukin (IL)-6, are elevated during the acute phase of KD and are associated with activation of vascular endothelial cells, which can produce varying degrees of vascular wall inflammation (Zhou et al., 2018; Li et al., 2019). TNF-α, IL-6, and other cytokines stimulate endothelial cells to upregulate matrix metalloproteinases (MMPs), leading to degradation of extracellular matrix (ECM) proteins and disruption of the vessel wall, thereby promoting the development of CALs in KD patients (Tian et al., 2022). Studies showed that TNF-α induces systemic vasculitis with high E-selectin expression associated with NF-κB activation in vascular endothelial cells during acute KD (Zakeri et al., 2000; Zhang et al., 2024). Previous work reported that 1,25-(OH)_2_D_3_ exerts anti-inflammation effects (El-Sharkawy and Malki, 2020; Ao et al., 2021). *In vitro* studies indicate that 1,25-(OH)_2_D_3_ may inhibits TNF-α-induced NF-κB activation, and the expression of E-selectin, intercellular adhesion molecule-1 (ICAM-1) and vascular cell adhesion molecule-1 (VCAM-1) in human microvascular endothelial cells, human umbilical vein endothelial cells, and human coronary arterial endothelial cells (Equils et al., 2006; Martinesi et al., 2006; Kudo et al., 2012). In addition, VDRs have been identified in coronary arterial endothelial cells (Kamboj et al., 2025). Together, these findings suggested that vitamin D adjuvant therapy can modulate the coronary inflammatory response in KD at the cellular level.

### 5.2 Endothelial function

Endothelial dysfunction in KD patients has been confirmed (Lo et al., 2023). EPCs are precursors of mature endothelial cells and are essential for maintaining arterial endothelium structure and function (He et al., 2004). Xu et al. found that EPCs function was significantly reduced in KD patients, suggesting that a relative EPCs deficiency may contribute to CALs (Xu et al., 2010). Therefore, agents that increase circulating EPCs could offer a novel therapeutic approach for KD. Clinically, VDR expression on circulating EPCs is markedly reduced in patients with coronary artery disease (Ai et al., 2018). *In vitro*, 1,25-(OH)_2_D_3_ (0.1–10 nM) increased directional migration and promoted adhesion-junction formation in endothelial colony-forming cells, while reducing TNF-α–induced endothelial barrier disruption (Schröder-Heurich et al., 2019). Together, these results indicate that vitamin D may limit vascular injury by enhancing EPC adhesion and migration (Hou et al., 2020). MicroRNA-27b (miR-27b) is reported to be upregulated in KD serum and to suppress human umbilical vein endothelial cell proliferation and migration by targeting and upregulating Smad family member 7 (Smad 7), a negative regulator of the TGF-β pathway. By inhibiting TGF-β/Smad signaling and subsequent endothelial-to-mesenchymal transition (EndoMT), miR-27b helps preserve the endothelial phenotype and prevent pathological vascular remodeling (Rong et al., 2018). A recent study from non-KD settings found that vitamin D/VDR signaling induces miR-27b expression (Ge et al., 2020). Collectively, vitamin D may enhance endothelial function, preserve vascular integrity, and reduce coronary artery damage risk in KD patients via the miR-27b/Smad7/TGF-β axis.

### 5.3 Platelet function

**FIGURE 1:**
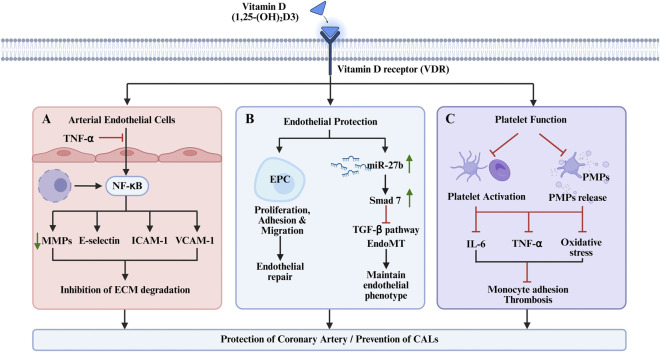
Protective mechanisms of vitamin D against vascular injury in Kawasaki disease. In Kawasaki disease (KD), proinflammatory cytokines (TNF-α, IL-6) initiate a cascade of vascular injury. **(A)** Vitamin D binds the vitamin D receptor (VDR) and inhibits NF-κB activation, which downregulates adhesion molecules (E-selectin, ICAM-1, VCAM-1) and matrix metalloproteinases (MMPs) to prevent extracellular matrix (ECM) degradation. **(B)** Vitamin D promotes endothelial progenitor cell (EPC) proliferation, adhesion, and migration, and activates the miR-27b/Smad7 axis to suppress TGF-β–mediated endothelial-to-mesenchymal transition (EndoMT), thereby enhancing endothelial repair. **(C)** Vitamin D reduces platelet hyperreactivity and the release of platelet-derived microparticles (PMPs), which inhibits monocyte adhesion and thrombosis. Collectively, these pathways act to lower the risk of coronary artery lesions (CALs) in KD. Created with BioRender.com.

Platelets are traditionally implicated in hemostasis and thrombosis, but their roles in immune responses and inflammation have received increasing attention (Mandel et al., 2022). Platelet hyperreactivity is also a hallmark of KD. Endothelial injury in the coronary arteries during KD can trigger platelet activation. Platelet-derived microparticles (PMPs) are vesicles shed from the plasma membrane of activated platelets (Varela-López et al., 2025). In KD, PMPs can damage endothelial cells and induce release of various inflammatory mediators, including adhesion molecules and cytokines (IL-1β, IL-7, IL-11). In children with KD, PMPs deliver arachidonic acid to endothelial cells, which upregulates ICAM-1 and thereby promotes monocyte adhesion (Jin et al., 2019). Kucukay et al. reported that platelet counts and mean platelet volume (MPV) were reduced following vitamin D treatment (Kucukay and Alanli, 2021). It has been hypothesized that vitamin D deficiency may promote the release of proinflammatory cytokines, including IL-6 and TNF-α, in patients with congestive heart failure (Schleithoff et al., 2006). These cytokines promote oxidative stress, which contributes to platelet activation. As noted above, 1,25-(OH)_2_D_3_ also decreases VCAM-1 expression. Thus, vitamin D may inhibit platelet and consequently reduce fibrinolysis, thrombosis, and inflammation in KD patients. Potential mechanism of the effect of vitamin D on Kawasaki disease is shown in Figure 1.

## 6 Clinical point of view

### 6.1 Serum 25-(OH)D_3_ in KD: clinical evidence and implications

More than a decade ago, researchers began to investigate whether vitamin D could serve as an adjuvant treatment for KD. As noted above, Kudo et al. (2012) showed *in vitro* that 1,25-(OH)_2_D_3_ has strong anti-inflammatory effects on human vascular endothelial cells, implying a potential immunomodulatory role during the vasculitis phase of KD. Subsequent clinical studies have corroborated a robust association between vitamin D levels and KD.

Authoritative evidence links vitamin D deficiency to KD risk. Zhang et al. (2024) performed a meta-analysis of 22 studies and reported that serum 25(OH)D_3_ levels were markedly lower in patients with KD than in healthy controls. The initial pooled effect size was an SMD of −0.94 (95% CI: −1.72 to −0.15, *P* = 0.019). After excluding one outlier, the SMD increased to −1.30 (95% CI: −2.05 to −0.55, *P* < 0.001), although substantial heterogeneity remained (*I* ^*2*^ = 98.26%). This heterogeneity could reflect unmeasured sources such as assay methodology and season of blood sampling. Sensitivity analyses and permutation testing (*P* = 0.007) nevertheless supported the robustness of the finding, indicating that the pronounced heterogeneity does not invalidate the primary conclusion. Furthermore, Okazaki et al. (2022) reported a strong inverse association between serum 25(OH)D_3_ concentration and KD risk, with an odds ratio of 29.4 for severe deficiency. Earlier studies by Stagi, et al. (2016) and Chen and Li (2014) corroborated marked deficiency and showed that vitamin D levels closely correlate with proinflammatory cytokines, including IL-6.

Findings are inconsistent on whether vitamin D status directly affects CALs development. Zhang et al. (2024) reported no clear association in pooled data (*P* > 0.05). By contrast, several single-center retrospective studies reported significant relationships: Stagi et al. (2016) linked 25(OH)D_3_ levels to coronary artery aneurysm occurrence (*P* = 0.005); Rigante et al. (2024) identified vitamin D < 30 ng/mL as an independent predictor of long-term cardiovascular abnormalities; Zhang et al. (2016) and Chen et al. (2022) found significantly lower serum 25(OH)D_3_ in KD patients with CALs than in those without. This divergence between pooled and single-center results suggests that vitamin D’s role in CALs progression is more complex than currently appreciated and may be modified by ethnicity, disease heterogeneity, or other unmeasured factors.

Vitamin D status is also linked to treatment resistance and changes during the convalescent phase. Jun et al. (2017) found in a Korean cohort that 25(OH)D_3_ < 20 ng/mL was significantly associated with increased risk of IVIG resistance (*P* = 0.023). Zhang et al. (2016) reported a consistent finding, showing that lower vitamin D levels correlated with a higher likelihood of IVIG nonresponse. Chen et al. (2022) observed that vitamin D levels in KD patients rose after IVIG compared with pre-treatment values, suggesting an association between IVIG therapy and changes in vitamin D levels.

Recent clinical trials have reinforced the therapeutic value of vitamin D supplementation. In a randomized controlled trial, Cheng et al. (2025) found that adding vitamin D to standard IVIG and aspirin reduced mean time to defervescence from 50.4 h to 27.2 h (*P* < 0.001) and decreased inflammatory markers, including white blood cell count, C-reactive protein, and platelet count. These results provide strong empirical support for vitamin D as an adjunctive therapy during the acute phase of KD. Although these preliminary results are encouraging, the trial used surrogate endpoints (time to defervescence and inflammatory markers) and was conducted at a single center, so definitive evidence that vitamin D supplementation reduces IVIG resistance or CALs in KD remains lacking. A summary of key clinical studies and their evidence levels is presented in Table 1.

**TABLE 1:** Study assessed levels of vitamin D in kawasaki disease patients.

Author, year	Country	Study design	Age (Years)	Case (male)	Control (male)	Vitamin D marker	Main finding	Level grade
Zhang et at. (2024)	China	Meta-analysis	2.8 ± 0.8	N/A	N/A	25-(OH) D_3_	✓Reduced serum vitamin D is significantly associated with KD occurrence ✓Vitamin D alone cannot predict CAL in KD.	Level I
Okazaki (2022)	Japan	Case–control	0.2–10.4	86 (50)	290 (166)	25-(OH) D_3_	✓Vitamin D deficiency predicts KD onset (OR = 4.9–29.4) ✓Low vitamin D is not associated with IVIG resistance or CAL.	Level III (H)
Stagi et al. (2016)	Italy	Case–control	1.4–7.5	73 (58)	234 (149)	25-(OH) D_3_	✓KD patients have severely lower 25-(OH) D_3_ levels than controls. ✓25-(OH) D_3_ might contribute to coronary artery damage in KD.	Level III (H)
Chen and Li (2014)	China	Case–control	0.3–4	35 (26)	50 (27)	25-(OH) D_3_	✓Serum 25-(OH)D_3_ level significantly increased after IVIG in KD. ✓Pre-treatment 25-(OH) D_3_ was positively correlated with IL-6.	Level III (M)
Rigante et al. (2024)	Italy	Retrospective Cohort	0.2–7.3	65 (42)	N/A	25-(OH) D_3_	✓Vitamin D deficiency is an independent risk factor for CVAs in KD.	Level III (H)
Zhang et al. (2016)	China	Case–control	3.3 ± 1.7	242 (138)	80 (45)	25-(OH) D_3_	✓Decreased 25-(OH)D_3_ level is associated with an increased risk of CAL. ✓Low 25-(OH)D_3_ associated with IVIG non-response	Level III (H)
Chen (2022)	China	Case–control	0.5–4	200 (121)	35 (19)	1,25 (OH) _2_ D_3_	✓Serum 1,25-(OH)_2_D_3_ level is decreased in children with KD. ✓Low 1,25-(OH)_2_D_3_ predicts higher CAL incidence.	Level III (H)
Jun (2017)	Korea	Case–control	3.1 ± 1.7	39 (18)	52 (38)	25-(OH) D_3_	✓Low vitamin D is a risk factor for IVIG resistance in KD. ✓No significant association with CAL.	Level III (H)
Cheng et al. (2025)	China	RCT	1–5	60 (32)	60 (30)	N/A	✓Vitamin D (400 IU/day) reduces fever, IVIG non-response, and inflammation in KD. ✓Vitamin D has no significant preventive effect on CAL.	Level II

^a^ Level of evidence was graded using the OCEBM, criteria: Level I, systematic reviews or meta-analyses of all relevant high-quality RCTs, or large-sample multicenter RCTs; Level II, results from a single large-sample RCT; Level III, case–control studies and cohort studies. The OCEBM, criteria are provided in Supplementary Appendix S2.

^b^ Study quality for cohort and case–control studies was assessed using the NOS, which evaluates three domains: selection of study groups, comparability of groups, and ascertainment of exposure or outcome. The total NOS, score ranges from 0 to 9; scores of 7–9 denote high quality, 4–6 denote moderate quality, and <4 denote low quality. The NOS, scale is provided in Supplementary Appendix S3.

KD: kawasaki disease; CAL: coronary artery lesions; CVAs: Cardiovascular abnormalities; IVIG: intravenous immunoglobulin; IL-6: Interleukin-6; OCEBM: Oxford Centre for Evidence-Based Medicine; RCTs: Randomized controlled trials; NOS: Newcastle-Ottawa Scale; H: High-quality evidence; M: Moderate-quality evidence.

When clinical suspicion of vitamin D deficiency arises in children with KD, it should be assessed and treated following standard pediatric guidelines. Vitamin D supplementation aimed at reducing IVIG resistance or coronary artery lesions remains investigational and should not be used routinely until multi-center prospective trials with hard clinical endpoints confirm its benefit. Serum 25(OH)D_3_ may be measured, but levels should not be used for risk stratification of adverse KD outcomes.

### 6.2 Potential molecular mechanism of 1,25(OH)_2_D_3_ in kawasaki patients

Recent studies have examined the molecular effects of vitamin D in KD (Qi et al., 2017; Qi et al., 2017; Zhou et al., 2022). Abnormal T cell activation plays an important role in KD pathogenesis (Wakiguchi et al., 2015). In peripheral blood T cells from children with KD, 1,25-(OH)_2_D_3_ (10^−7^–10^−3^ μM, 24 h) inhibited ERK1/2 phosphorylation and restored P53 protein expression, thereby modulating the balance between T cell proliferation and apoptosis (Qi et al., 2017). P53 is a transcription factor, found in the nucleus and cytoplasm, that binds DNA and regulates many genes (Wei et al., 2006); its activation promotes apoptosis. The absence of P53 protein expression T cells of KD patients indicates that T cell apoptosis is suppressed in these patients. The ERK family, a subset of mitogen-activated protein kinases (MAPKs), comprises two main isoforms, ERK1 and ERK2. Activated ERK translocates to the nucleus, activates transcriptional regulators, initiates gene transcription, and promotes cell proliferation (Liu et al., 2013). ERK pathway activation therefore enhances T cell growth, proliferation, and differentiation in KD. Qi and colleagues reported that *in vitro* treatment with 1,25(OH)_2_D_3_ inhibited ERK1/2 signaling and restored P53 protein activation in KD T cells. They propose that 1,25-(OH)_2_D_3_ regulates T lymphocyte proliferation in KD by activating P53 and inhibiting the ERK1/2 pathway (Qi et al., 2017).

**FIGURE 2:**
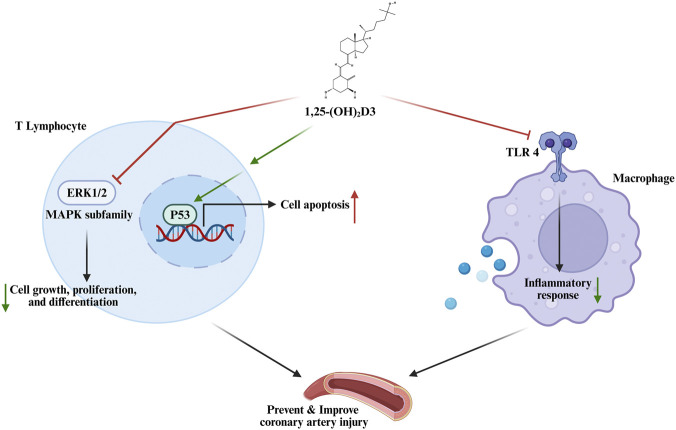
Potential signaling pathways of 1,25-(OH)_2_D_3_ in Kawasaki disease. Left: In T lymphocytes, 1,25-(OH)_2_D_3_ activates p53 and inhibits the ERK1/2 signaling pathway, thereby modulating the balance between apoptosis and proliferation and preventing excessive T cell expansion and differentiation. Right: In macrophages, 1,25-(OH)_2_D_3_ reduces the KD serum–induced inflammatory response by inhibiting the TLR4 signaling pathway. By these dual regulatory actions, 1,25-(OH)_2_D_3_ helps prevent and mitigate coronary artery injury in Kawasaki disease. Created with BioRender.com.

TLR4 pathway is central to macrophage-mediated defense against external pathogens, but its overactivation contributes to inflammatory tissue damage. Inhibition of TLR4 signaling has been shown to attenuate inflammatory responses and to protect myocardium (Vaez et al., 2023). In macrophages stimulated with KD serum, TLR4 expression was markedly elevated, and 1,25-(OH)_2_D_3_ (1–100 nmol/L, 48 h pretreatment) substantially suppressed this elevation. (Zhou et al., 2022). They proposed that supplementation with 1,25-(OH)_2_D_3_ can inhibit KD serum-induced inflammatory response via the TLR4 signaling pathway and thereby prevent or ameliorate coronary artery injury in children with KD. Potential signaling pathways of 1,25(OH)_2_D_3_ in KD is shown in Figure 2.

## 7 Summary

Existing evidence indicates a significant association between vitamin D deficiency and the occurrence of KD, but it does not establish causality. Studies report that serum 25(OH)D_3_ levels in children with KD are significantly lower than those in healthy peers. Low 25(OH)D_3_ concentrations are linked to two adverse clinical outcomes: increased resistance to IVIG therapy and a higher incidence of CALs.

Mechanistically, vitamin D confers protection through several pathways: it inhibits NF-κB signaling in endothelial cells, thereby reducing coronary artery inflammation; it suppresses TLR4-mediated inflammatory responses in macrophages; it promotes endothelial repair by enhancing EPCs function; and it modulates T cell survival via the P53/ERK pathway to limit immune-mediated injury. These anti-inflammatory and immunomodulatory actions provide a plausible biological basis linking vitamin D deficiency to higher risks of IVIG resistance and CALs.

## 8 Limitations

This review has several limitations. First, as a narrative rather than systematic review, it is susceptible to potential bias in study selection and interpretation. To mitigate this, two independent investigators performed the literature search. Second, most available studies on vitamin D and KD are small-scale and observational, which limits statistical power for detecting weak associations and precludes causal inference. Third, substantial methodological heterogeneity exists across studies, including the use of different vitamin D forms (e.g., vitamin D_2_, D_3_, and their metabolites) and a lack of consensus on cutoff values for defining vitamin D deficiency or insufficiency in KD, complicating cross-study comparisons and synthesis. Fourth, many mechanistic insights are derived from *in vitro* experiments, animal models, or non-KD populations, which may not fully reflect the pathophysiology of human KD. Finally, much of the evidence is derived from East Asian pediatric populations, which limits the generalizability of our findings to other ethnic or geographic groups. Therefore, the conclusions drawn primarily from East Asian cohorts should be applied cautiously to non-Asian populations.

Future research should address two priority questions. First, prospective cohort studies and randomized controlled trials in diverse geographic and ethnic settings are needed to determine whether low vitamin D levels are a risk factor for KD or a consequence of the disease process; Second, randomized controlled trials should evaluate whether vitamin D supplementation as an adjunctive therapy in patients with KD reduces IVIG resistance and the incidence of CALs, while also assessing safety. Only through globally high-quality studies can we determine whether optimizing vitamin D status improves clinical outcomes in children with KD.
