# Supplementary material for: Vitamin D and Kawasaki disease
Source: Front Pharmacol. 2026 Jun 2;17:1851161. doi: 10.3389/fphar.2026.1851161 (PMC13269355; doi:10.3389/fphar.2026.1851161)
Supplement: Supplementary file 1 [file Supplementaryfile1.docx]

**Appendix 1.**

**Summary of evidence strength grading**

| **Category** | **Study** | **Study Type** | **Key Finding** | **Level of Evidence** |
| --- | --- | --- | --- | --- |
| **Guidelines & Consensus** | Global Consensus on 25(OH)D (2024) | International consensus | 25(OH)D₃ as gold standard for vitamin D assessment. | **I** |
|  | AHA Scientific Statement (Jone et al., 2024) | Scientific statement | KD is the leading cause of acquired heart disease in children. | **I** |
|  | JCS/JSCS Guideline (Fukazawa et al., 2020) | Clinical guideline | Standardized diagnosis and management of cardiovascular sequelae in KD. | **I** |
| **Meta-analyses** | Zhang et al., 2024 | Meta-analysis (22 studies) | Serum 25(OH)D₃ markedly lower in KD vs. controls (SMD −1.30; P < 0.001). | **I** |
|  | Zheng et al., 2021 | Meta-analysis | IVIG resistance associated with higher CAL risk. | **I** |
| **Large Epidemiological Studies** | Yang et al., 2020 | Nationwide survey (N = 460,537) | Vitamin D deficiency 6.7%, insufficiency 15.9% in Chinese children. | **II** |
|  | Hilger et al., 2014 | Systematic review (195 studies, 44 countries) | 37.3% of studies reported mean 25(OH)D < 50 nmol/L. | **II** |
|  | Fukazawa et al., 2020 | Epidemiological study | KD prevalence highest in Asian children (Japan: 319.6/100,000). | **II** |
| **Single-center Clinical Studies** | Cheng et al., 2025 | RCT (single-center) | Vitamin D reduced time to defervescence (50.4→27.2 h; P < 0.001) and inflammatory markers. | **II** |
|  | Okazaki et al., 2022 | Case-control | Severe vitamin D deficiency and KD risk (OR = 29.4). | **III** |
|  | Stagi et al., 2016 | Case-control | 25(OH)D₃ levels correlated with coronary aneurysm (P = 0.005). | **III** |
|  | Rigante et al., 2024 | Cohort | Vitamin D < 30 ng/mL as independent predictor of long-term cardiovascular abnormalities. | **III** |
|  | Jun et al., 2017 | Case-control | 25(OH)D₃ < 20 ng/mL associated with IVIG resistance (P = 0.023). | **III** |
| **In Vitro Studies** | Kudo et al., 2012 | In vitro | 1,25-(OH)₂D₃ inhibited TNF-α-induced ICAM-1 and VCAM-1 expression. | **V** |
|  | Equils et al., 2006 | In vitro | 1,25-(OH)₂D₃ inhibited TNF-α-induced NF-κB activation and E-selectin expression. | **V** |
|  | Qi et al., 2017 | In vitro | 1,25-(OH)₂D₃ modulated P53/ERK pathway in KD T cells. | **V** |
|  | Zhou et al., 2022 | In vitro | 1,25-(OH)₂D₃ suppressed TLR4 expression induced by KD serum. | **V** |
| **Animal Model** | Chen et al., 2025 | Mouse model | 25(OH)D₃ levels predicted CAL risk in a mouse model of KD. | **V** |
| **Cross-disease Extrapolations** | Kucukay et al., 2021 | Clinical study (non-KD) | Vitamin D reduced platelet count and MPV. | **V** |
|  | Schleithoff et al., 2006 | Clinical study (CHF) | Vitamin D deficiency promoted IL-6 and TNF-α release. | **V** |

*Levels of evidence are based on the Oxford Centre for Evidence-Based Medicine (OCEBM) criteria. See Appendix 2 for detailed definitions of each level.

**Appendix 2.**

**Oxford Centre for Evidence-Based Medicine**

| **Level I** | Systematic reviews or meta-analyses of all relevant high-quality randomized controlled trials (RCTs), or large-sample multicenter RCTs.  large-sample multicenter randomized controlled trials (RCTs). |
| --- | --- |
| **Level II** | A single large-sample RCT. |
| **Level III** | Case–control studies and cohort studies. |
| **Level IV** | Uncontrolled case series. |
| **Level V** | Expert opinion, descriptive studies, case reports. |

**Appendix 3.**

**Newcastle-ottawa quality assessment of case-control studies included in the review**

| Author | Selection | | | | Comparability | Exposure | | | Total  scores |
| --- | --- | --- | --- | --- | --- | --- | --- | --- | --- |
|  | Case definition adequate | Representativeness of the cases | Selection of controls | Definition of controls | Comparability of cases and controls | Ascertainment of exposure | Same method of ascertainment | Non-Response rate |  |
| [Okazaki](https://pubmed.ncbi.nlm.nih.gov/?term=Okazaki+N&cauthor_id=35831250), 2022 | * | * | * | * | * | * | * | * | 8 |
| Stagi, 2016 | * | * | * | * | ** | * | * | * | 9 |
| Chen, 2014 | * | * | * | * | - | * | * | - | 6 |
| Rigante, 2024 | * | * | * | * | * | * | * | * | 8 |
| Zhang, 2016 | * | * | * | * | * | * | * | * | 8 |
| Chen, 2022 | * | * | * | * | - | * | * | * | 7 |
| Jun, 2017 | * | * | * | * | - | * | * | * | 7 |

Note: A study can be awarded a maximum of one star for each numbered item within the Selection and Exposure categories. A maximum of two stars can be given for Comparability.

The Newcastle-Ottawa Scale (NOS) for assessing the quality of nonrandomised studies in meta-analyses, 2011. Availabe online: www.ohri.ca/

**Newcastle-ottawa quality assessment of cohort studies included in the review**

| Author | Selection | | | | Comparability | Exposure | | | Total  scores |
| --- | --- | --- | --- | --- | --- | --- | --- | --- | --- |
|  | Representativeness | Non-exposed selection | Ascertainment of exposure | Outcome absent at baseline | Comparability of controlled for confounders | Assessment of outcome | Adequate follow-up duration | Follow-up completeness |  |
| Rigante, 2024 | * | * | * | * | * | * | * | * | 8 |

Note: A study can be awarded a maximum of one star for each numbered item within the Selection and Exposure categories. A maximum of two stars can be given for Comparability.

The Newcastle-Ottawa Scale (NOS) for assessing the quality of nonrandomised studies in meta-analyses, 2011. Availabe online: www.ohri.ca/
